# Efficacy of Comedy on Health-Related Quality of Life and Oxidative Stress in Cancer Survivors

**DOI:** 10.7759/cureus.42760

**Published:** 2023-07-31

**Authors:** Ryo Sakamoto, Yukariko Hida, Mariko Shiozaki, Hiroko Motooka, Atsuko Koyama

**Affiliations:** 1 Psychosomatic Medicine, Kindai University Faculty of Medicine, Osakasayama City, JPN; 2 Palliative Care, Centre for Palliative Care, Kindai University Hospital, Osakasayama City, JPN; 3 Psychology, Kindai University Faculty of Applied Sociology, Higashiosaka City, JPN

**Keywords:** comedy, oxidative stress index, health care seeking behaviours, cancer survivorship, quality of life (qol)

## Abstract

Introduction: Cancer survivors have reduced health-related quality of life (HRQOL) due to impaired daily functioning. In addition, daily stress leads to worsening oxidative stress. The purpose of this study is to investigate the efficacy of laughter therapy on HRQOL and oxidative stress in cancer survivors.

Methods: This before-and-after study asked cancer survivors to watch a 15-minute or longer comedy video over a four-week period to assess the Functional Assessment of Cancer Therapy-General (FACT-G), EuroQOL 5 dimension 3-level (EQ-5D-3L), Hospital Anxiety and Depression Scale (HADS), biological Antioxidant Potential (BAP), Reactive Oxygen Metabolites-derived compounds (d-ROMs), Oxidative Stress Index (OSI), and the antioxidant/oxidative stress ratio.

Results: The nonparametric Friedman test showed significant increases from baseline in FACT-G and EQ-VAS scores and significant decreases in HADS-Anxiety and HADS-Depression scores. Post hoc analyses showed that these items commonly differed significantly at baseline versus three and four weeks after Bonferroni correction. T-test results in the biological analysis revealed small and moderate effects with significant differences in BAP (p < 0.01, d = 0.49), OSI (p = 0.03, d = 0.33), and BAP/d-ROMs (p < 0.01, d = 0.51).

Conclusion: These results suggest that daily comedy viewing may be an effective intervention to improve quality of life and antioxidant capacity in cancer survivors. Considering its safety, convenience, and low cost, it should be considered a high-value intervention for cancer survivors.

## Introduction

Recent advances in cancer treatment have increased the five-year survival rate to 80% or 90%, depending on the type of cancer, and this trend is the same worldwide [1]. Cancer is changing from an incurable disease to a chronic disease that survivors live with for a long time. The second Basic Plan for the Promotion of Cancer Control, which was launched in Japan in 2012, clearly states "building a society where people can live with peace of mind even after cancer" as one of its overall goals [2]. From this perspective, it is important not only to aim for prevention and the development of medical technology and treatment but also to improve long-term life quality after cancer diagnosis and treatment. However, cancer survivors must live with various burdens [3] and fears of recurrence [4]. And the health-related quality of life (HRQOL) of cancer survivors has been reported to be diminished owing to the negative impact on daily functioning and quality of life [5].

Worsening HRQOL can lead to decreased feelings of health and well-being and a vicious cycle of behaviors. Oxidative stress is also increased with daily stress, leading to the development of neurological and psychiatric diseases [6]. Oxidative stress is also an important factor in the development and progression of cancer [5]. Laughter therapy has been reported to reduce levels of depression, anxiety, and stress in cancer patients [7]. However, there are few studies reporting the effect of laughter therapy on HRQOL and oxidative stress, and there have been no previous studies investigating changes in HRQOL and oxidative stress among cancer survivors owing to watching comedy videos. Furthermore, although several categorizations of laughter therapy have been presented, no clear definition has been established. In the present study, we used a simple, non-invasive approach with the aim of developing an effective option to help improve HRQOL among survivors of cancer in the future.

## Materials and methods

Study design

This was a single-arm study to evaluate the effects of comedy therapy on HRQOL and oxidative stress among cancer survivors. Before carrying out this study, the protocol was registered in R000051259 of the university hospital medical information (UMIN) clinical trials registry (CTR) (https://center6.umin.ac.jp/cgi-open-bin/ctr/ctr_view.cgi?recptno=R000051259). This study was conducted in accordance with the Declaration of Helsinki and the Japanese Ethical Guidelines for Clinical Research. Written informed consent was obtained from all participants. The protocol of this study was approved by the Ethics Committee of Kindai University School of Medicine (R03-119).

Study participants

The study population comprised adult patients diagnosed with cancer whose cancer was currently cured or stable. Eligibility criteria were: written informed consent, male or female sex, age 20 years or older, diagnosed with cancer, and cancer status cured or stable. Exclusion criteria included treatment with anticancer drugs; however, patients taking hormone therapy to prevent recurrence (e.g., in breast cancer or prostate cancer) were included. Additional exclusion criteria were patients undergoing treatment for cancer recurrence or metastasis, patients whose condition was unstable, patients on contraceptive pills, and patients in the terminal stage of cancer. We also excluded patients who, in the opinion of the investigator, were inappropriate for inclusion in the study. For example, we excluded patients with a mental illness that prevented them from making informed decisions or those with severe late effects or sequelae of treatment that made it difficult for the patient to participate in this study. Participants in this study received approximately $3 in e-money and their own d-ROMs and BAP results.

Study intervention

We asked patients to watch a comedy video on a previously prepared DVD for at least 15 minutes each day. The content of the comedy is manzai: Japanese stand-up comedy with a straight man and a funny man, and rakugo; traditional Japanese comic storytelling. The DVD was recorded to allow for 15 minutes of viewing each day during this study period. After watching the DVD, we asked participants to check a box on a Google form or on a paper form distributed on the first day of the intervention. If the participant was taking medication for a pre-existing condition, the medication could be continued unless it was included within the exclusion criteria. The trial was not powered to determine whether participants consumed dietary or supplemental antioxidants during the intervention period. If the participant wished to discontinue the study, all video and psychological test materials in the participant's possession were to be returned by mail or in-person to the research office.

Study procedure

Participants were recruited via the web and print media in cooperation with the public relations department of Kindai University. The advertisement included the schedule and outline of the study, and the research office accepted applications for participation by e-mail. After receiving the application, the research office contacted each participant by phone and e-mail to inform them of the appointment date, time, and meeting place.

Prior to participation in the study, the principal investigator obtained informed consent from all participants. Questionnaires on demographic characteristics, as well as psychological tests, were completed when participants visited Kindai University Hospital on day 1 of the study and signed the informed consent form. Participants were asked to answer the questions in front of the principal investigator. For the psychological tests, we provided participants with the necessary materials for the second and subsequent tests, together with a self-addressed envelope, on day 1 (baseline: T1). We asked participants to complete the form at one week (T2), two weeks (T3), and three weeks (T4) and then send the completed form to the research secretariat. If a participant was unable to watch the DVD for any reason, we asked them to note this on the test form and send the form to us after completing the administration confirmation. We asked participants to give the four-week (T5) portion of the questionnaire to the person in charge when they came to the hospital. The Ten-Item Personality Inventory Japanese Version (TIPI-J) was administered on T1 only so as to obtain demographic characteristics.

For biological analyses, 40 µL of blood was collected from the fingertip using a Safe-T Pro Plus Lancet. The sample was stored in a blood collection Microvette at 2-8 °C, and measurements were taken on the day of collection using a free radical analyzer.

The principal investigator provided each participant with three comedy DVDs on loan. The loan period was through T5, and participants were asked to return the DVDs when they visited Kindai University Hospital. If the study was canceled, the participants were asked to mail or return all DVDs to the principal investigator via cash on delivery. This study schedule is shown in Table 1.

**Table 1:** Study schedule. Post questionnaire: Stress in daily life before and after the COVID-19 pandemic. Borrowed comedy DVD preferences. Presence or absence of major life events during the study period.

Implementation items	Baseline	Follow-up							
	T1	T1 to T2	T2	T2 to T3	T3	T3 to T4	T4	T4 to T5	T5
Demographics	X								
Health-Related Quality of Life (FACT-G)	X		X		X		X		X
EuroQOL 5 dimension 3-level (EQ-5D-3L)	X		X		X		X		X
Hospital Anxiety and Depression Scale (HADS)	X		X		X		X		X
Biological Antioxidant Potential (BAP)	X								X
Reactive Oxygen Metabolites-derived compounds (d-ROMs)	X								X
TIPI-J	X								
Post questionnaires									X
Intervention (comedy watching)	X	X	X	X	X	X	X	X	X
Confirmation of implementation	X	X	X	X	X	X	X	X	X

Description of psychological tests

The Functional Assessment of Cancer Therapy-General (FACT-G) is used to assess HRQOL in patients with cancer. This instrument comprises 27 items: seven physical, seven social/family, six psychological, and seven functional items. Subscale scores are added to obtain the total FACT-G score. The higher the score, the better the HRQOL. This instrument has been validated for reliability and validity [8]. This scale has demonstrated high internal consistency in past studies (α = 0.89) [8].

The EuroQOL 5-dimension 3-level (EQ-5D-3L) is used to comprehensively assess a patient's HRQOL and includes five questions addressing the level of mobility, personal care, usual activities, pain or discomfort, and anxiety or depression. Each dimension has three levels: no problems, some problems, and extreme problems. The instrument has been validated for reliability and validity [9]. The EQ-5D-3L index ranges from −0.59 to 1.0, with positive values indicating a perfect outcome and negative values implying states worse than death. Another part of the questionnaire includes a visual analog scale (EQ-VAS) that measures patients' self-perceived health status. The EQ-VAS rates participants' self-reported health status on a scale from 0 (poor health) to 100 (best health).

The Hospital Anxiety and Depression Scale (HADS) is used to measure psychiatric symptoms (anxiety and depression) in patients with physical illnesses. The HADS has 14 items, seven for depression and seven for anxiety. A score of 0-7 on either the HADS(A) or HADS(D) indicates no anxiety or depression; a score of 8-10 on either the HADS(A) or HADS(D) indicates possible anxiety or depression; and a score of ≥11 indicates definite anxiety or depression. The reliability and validity of this scale have been confirmed internationally [10]. Previous analyses reported good internal consistency of both scales (α > 0.80) [10].

The TIPI-J is used to assess a patient's Big Five personality traits and comprises 10 questions examining extraversion, cooperation, industriousness, neuroticism, and openness. The instrument has been validated for reliability and validity [11]. Each scale demonstrated high internal consistency (i.e., extraversion (α = 0.92), agreeableness (α =0.85), conscientiousness (α = 0.82), emotional stability (α = 0.91), and openness to experiences (α = 0.86) [11].

Additional questionnaires were administered four weeks after baseline to assess the following: stress in daily life before and after the COVID-19 pandemic (reported on a Likert scale of 10 = highest stress, 0 = no stress at all); whether the comedy DVDs borrowed in this study met their own preferences (reported on a Likert scale of 10 = highest preference, 0 = no preference at all); whether a major life event occurred during the study period (e.g., death in the family, divorce, separation, new illness, etc.; reported as yes/no).

Biological analyses

Biological Antioxidant Potential

The Biological Antioxidant Potential (BAP) is an indicator of reducing power (antioxidant power), which is the degree to which iron oxide can be reduced, and this is measured via a redox reaction. When trivalent iron Fe^3+^ in the reagent is reduced to divalent iron Fe^2+^ using the reducing substance in the serum, the color of the solution becomes colorless, and the intensity of the color change is proportional to the reducing power in the serum; this is taken as a measure of the reducing power or antioxidant capacity in the sample [12].

Reactive Oxygen Metabolites-Derived Compounds

Assessment of oxidative stress requires accurate measurement of the level of excess free radicals produced in the body. However, free radicals are difficult to measure in vivo owing to their short lifespan and high reactivity. The d-ROMs test is a method to assess the level of oxidative stress in vivo by measuring hydroperoxides produced by free radicals and reactive oxygen species in vivo using a colorimetric reaction [13].

For the detection of d-ROMs, the reaction of N,N-diethyl-para-phenylenediamine with hydrogen peroxide in serum was measured according to the colorimetric change in absorbance at 505 nm using a specific free radical analyzer (FREE carpe diem, Wismerl Co., Ltd., Tokyo, Japan). The d-ROM values are expressed in colorimetric units based on the calculated ab absorbance. BAP levels in serum were also measured using the same instrument, and the reduction in FeCl3 was detected by the disappearance of a radish color [14]. The oxidative stress index (OSI) is calculated by multiplying the ratio of d-ROM/BAP by 100. Celi suggested that when assessing oxidative stress using ROMs and BAP, combining ROM and BAP data rather than treating them separately provides more accurate information about the level of oxidative stress [15]. The antioxidant/oxidative stress ratio was calculated using the equation: BAP/d-ROMs. The level of oxidative stress was evaluated according to the balance between oxidative level and antioxidant capacity, and the ratio of oxidative level to antioxidant capacity is often used as an indicator. Results of an analysis using (d-ROMs/BAP) × 8.85 as the OSI have been reported in the Japanese population [16]; we performed this analysis in the present study.

Sample size calculation

No study with a research design similar to that used in our present study was found for HRQOL and oxidative stress in cancer survivors. The clinically important point change on the FACT-G has been reported to be five points [17]. A study similar to ours used a combined intervention method of laughter, deep breathing, meditation, and music for patients with gynecological cancer. That study showed an improvement in FACT-G mean scores of 64.7 (standard deviation [SD] 11.6) before the intervention and 69.7 (SD 12.1) after the intervention [18]. Based on the results of that study, the effect size was 0.4151576 when calculated using Gpower. The present study was considered to have a similar effect to that previous study; when evaluated using the Mann-Whitney U test at a significance level of α = 0.05, the minimum number of cases required to achieve a power of 1 − β of 0.8 or greater was 41.

Statistical analyses

Differences between FACT-G, ED-5Q-3L, and HADS at the different time points (T1, T2, T3, T4, and T5) were tested with non-parametric Friedman's analysis of variance (ANOVA), and post-hoc analyses were performed. Post-hoc analysis p-values were corrected using the Bonferroni correction. BAP, d-ROMs, BAP/d-ROMs, and OSI scores were compared using the t-test. Statistical analyses were performed using IBM SPSS 27 (IBM Corp., Armonk, NY, USA). A p-value of < 0.05 was considered significant.

## Results

Fifty participants (10 males and 40 females) were included in the study. Additionally, three male and four female participants dropped out of the study before the four-week follow-up owing to scheduling or for other reasons. Table 2 summarizes the baseline characteristics of our study population. The overall median age was 55 (interquartile range [IQR], 51.5-64) years, and 83.7% were women. The diagnoses of participants were (in order of increasing frequency) breast, colorectal, and uterine cancer. In total, 48.8% of respondents reported having the habit of watching comedy. Participants reported stress levels changed from 5 (IQR, 3-5.5) before to 6 (IQR, 5-7) after the pandemic. Major life events occurred for 20.9% of participants in this study. The average response to whether the DVDs were to their liking was 6 (IQR, 3-8).

**Table 2:** Characteristics of our study population (n = 43).

Characteristics	No. (%)
Age, years	
Median (IQR)	55 (51.5–64)
Mean (SD)	58.7 (10.5)
Sex	
Male	7 (16)
Female	36 (84)
Married	31 (72.0)
Job, Employed	36 (83.7)
Cancer type (some with two onsets)	
Breast	19 (44.1)
Colorectal	8 (18.6)
Uterine	8 (18.6)
Lung	4 (9.3)
Other	8 (18.6)
Months after cancer onset, the median (IQR)	66 (38–132)
Hospital visit for non-cancer	23 (53.4)
The habit of watching comedy	21 (48.8)
Changes in stress in daily life after the COVID-19 epidemic	
COVID 19 before epidemic, median (IQR)	5 (3–5.5)
COVID 19 post epidemic, median (IQR)	6 (5–7)
TIPI-J domain	
Extraversion, median (IQR)	4.5 (4–6)
Agreeableness, median (IQR))	5.5 (4.5–6)
Conscientiousness, median (IQR)	3.5 (3–4.5)
Emotional stability, median (IQR)	4 (3–5)
Openness to experiences, median (IQR)	4.5 (3.5–5)
Major life events during this study	9 (20.9)
DVD preferences used in this study, median (IQR)	6 (3–8)

HRQOL, anxiety, and depression

**Figure 1:**
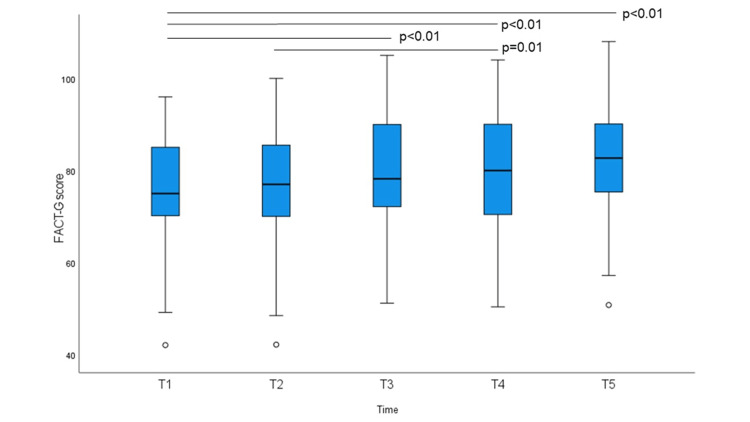
Functional Assessment of Cancer Therapy-General (FACT-G) total score at baseline and at weekly intervals. Bonferroni-corrected p-values in post-hoc analyses (Friedman test) are indicated (*p < 0.05).

**Figure 2:**
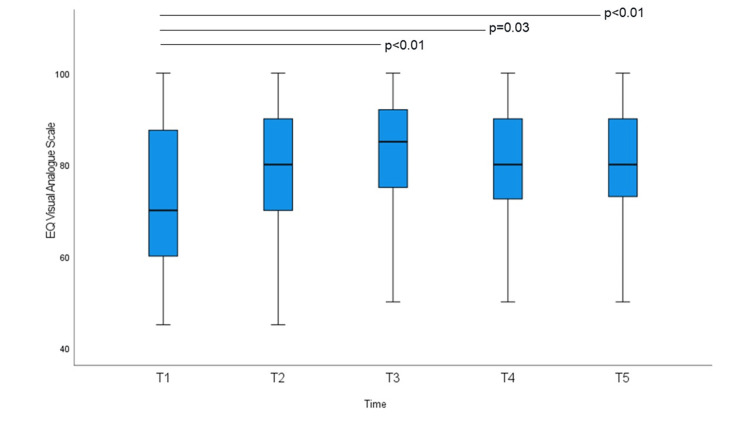
EuroQOL 5 dimension 3-level, with a visual analog scale (EQ-VAS), score at baseline and at weekly intervals. Bonferroni-corrected p-values in post-hoc analyses (Friedman test) are indicated (*p < 0.05).

**Figure 3:**
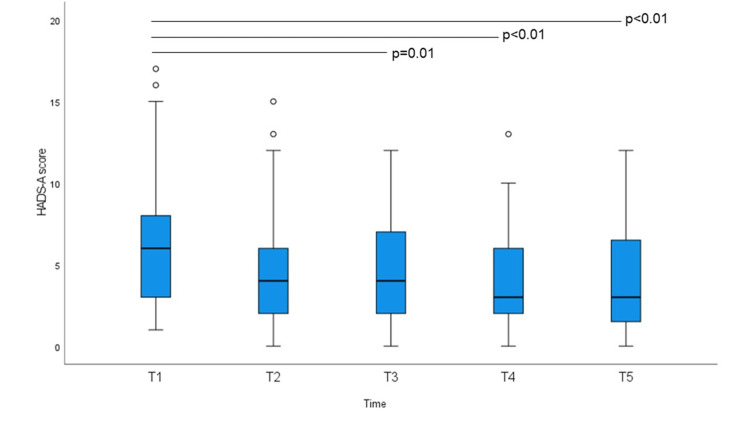
Hospital Anxiety and Depression Scale-anxiety (HADS-A) score at baseline and at weekly intervals. Bonferroni-corrected p-values in post-hoc analyses (Friedman test) are indicated (*p < 0.05).

**Figure 4:**
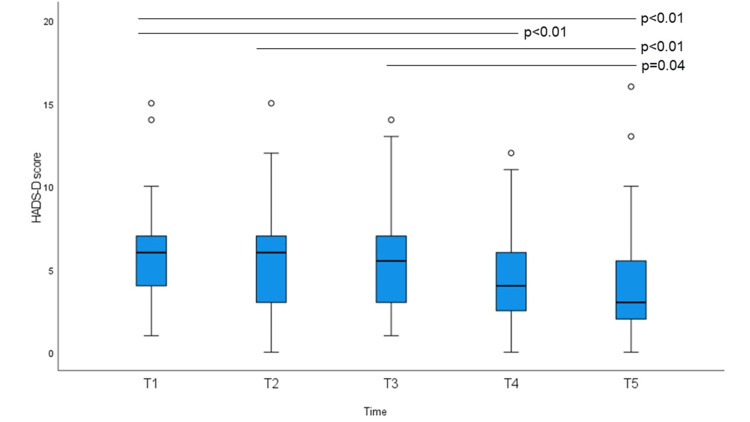
HAD-depression (HADS-D) score at baseline and at weekly intervals. Bonferroni-corrected p-values in post-hoc analyses (Friedman test) are indicated (*p < 0.05).

FACT-G, EQ-VAS, HADS-A, and HADS-D scores at baseline and at the four subsequent time points are shown in Figures 1-4. The non-parametric Friedman test revealed significant differences among the five-time points on the FACT-G, EQ-VAS, HADS-A, and HADS-D (χ2 (df = 4) = 30.493; p < 0.01, χ^2^ (df = 4) = 25.053; p < 0.01, χ^2^ (df = 4) = 30.735; p < 0.01, and χ^2^ (df = 4) = 27.813; p < 0.01, respectively). EQ-5D-3L index scores were not significantly different. Post-hoc analysis using Bonferroni correction showed significant differences for the FACT-G scores, which increased significantly on average from 76.11 at T1 to 79.95 at T3, 80.26 at T4, and 82.33 at T5. EQ-VAS scores increased significantly from 77.22 at T2 to 81.92 at T3, 80.48 at T4, and 81.39 at T5. HADS-A scores decreased significantly from 6.37 at T1 to 4.69 at T3, 3.97 at T4, and 4.06 at T5. HADS-D scores decreased significantly at T1 from 5.88 to 4.44 at T4 and 3.97 at T5; T2 from 5.57 to T5; and T3 and T5 also decreased significantly. There were no significant differences in the mean EQ-5D-3L scores.

BAP, d-ROMS, OSI, BAP/d-ROMs

A comparison between the means of all items in the biological analyses is shown in Table 3. Paired t-tests showed significant differences in the results for BAP (p < 0.01, Cohen's d = 0.49), the OSI (p = 0.03, Cohen's d = 0.33), and BAP/d-ROMs (p < 0.01, Cohen's d = 0.51), with small and moderate effects. The results for d-ROMs were not significantly different (p =0.79).

**Table 3:** Participants' levels of d-ROM, BAP, and OSI before and after the study. *Indicates p-value < 0.05.

Markers	Pre-mean (SD)	Post-mean (SD)	p-Value
d-ROMs (CARR Unit)	334.04 (79.04)	331.20 (88.88)	0.791
BAP (μmol/L))	1665.33 (653.44)	1892.59 (423.47)	0.003*
OSI	3.24 (4.98)	1.70 (0.73)	0.034*
ｄ-ROMs/BAP	5.11 (2.09)	5.98 (1.81)	0.002*

## Discussion

To the best of our knowledge, this was the first study to reveal changes in HRQOL and oxidative stress among cancer survivors as a result of viewing comedy. A strength of this study is that the findings suggest daily comedy viewing has the potential to improve HRQOL. Considering other findings of improved HRQOL in breast cancer survivors after eight weeks of rigorous exercise sessions [19], these results may lead to improved effectiveness in terms of a wider range of cancer patients, intervention duration, and location.

Another important aspect of this study is the implication of the results that daily comedy viewing may improve anxiety and depression. Prior studies have reported improvements in depression with mindfulness-based stress reduction (MBSR) in breast cancer survivors [20]; however, efficacy regarding anxiety has not been reported [21]. Laughter therapy has been found to reduce anxiety, albeit in non-cancer patients [22]. These results suggest that a daily habit of watching comedy may reduce anxiety as well as depression.

Of particular interest is the increase in antioxidant capacity among our participants. Laughing lowers the stress hormone cortisol [23]. More frequent laughter is associated with a more effective reduction in stress symptoms during stressful events [24]. Antioxidant capacity is also improved by oral supplementation with antioxidants [25] and lifestyle interventions such as yoga [26]. The results of this study and those of these previous studies suggest that the cause of the improved antioxidant capacity may be due to the effect of laughter as well as the habit of watching comedies, which may have led to lifestyle improvements. d-ROMs did not differ significantly. Older age and chronic illness are associated with higher d-ROMs [27,28]. In this study, the participants were relatively older, and approximately half of them had comorbidities other than cancer. It is possible that these factors did not result in as effective a response as antioxidant capacity.

The strength of this study is that it shows that daily comedy viewing may have beneficial effects on HQOL and oxidative stress in cancer survivors. However, study limitations include the following: first, this is a single-arm pilot study. Therefore, the results cannot be compared with those of the comparison and control groups. A randomized controlled trial should be designed and conducted based on these results. Second, post-hoc tests showed that effect sizes greater than 0.5, such as BAP, had power greater than 0.8, while the smallest effect size, OSI (r=0.33), was only 0.5, suggesting that the sample size may have been inadequate. Larger sample sizes should be used when other researchers conduct studies similar to this study. Third, cognitive biases may exist. The association between laughter and health has been widely reported in the media, and public expectations of the benefits of laughter may outweigh the actual effects [29]. In particular, we used a self-administered survey in this study; therefore, we cannot eliminate the effects of this bias. Fourth, there is the issue of potential confounding factors. The most important issue in this study is the region in which it was conducted. Osaka has long been a region where Japanese comedy viewing is popular, and it is possible that the participants were more receptive to the comedy DVDs provided in this study. It is also possible that the participants were more likely to continue watching comedy DVDs for 15 minutes or more each day compared to people in other parts of Japan. In future research, it will be necessary to recruit participants taking into account the region and their comedy viewing habits. Fifth, there may also be selection bias with respect to the sex distribution because approximately 80% of participants were women. The bias in the sex distribution may be due to the fact that men have higher employment rates than women and may have been less likely to participate in the study for this and other reasons. This may therefore affect the generalizability of the findings to men. Previous studies have reported that women tend to respond to laughter therapy to a greater extent than men [30], but we do not believe that this significantly affects the value of our results.

## Conclusions

In this study, we showed that daily comedy viewing may have beneficial effects on HQOL and oxidative stress in cancer survivors. Future randomized controlled trials based on this study may be able to demonstrate that daily comedy viewing may be an effective intervention to improve HRQOL and antioxidant capacity in cancer survivors. Comedy is safe, convenient, and inexpensive, making it an extremely valuable intervention for cancer patients.

## References

[1] Miller KD, Siegel RL, Lin CC (2016). Cancer treatment and survivorship statistics, 2016. CA Cancer J Clin.

[2] (2023). Ministry of Health, Labour and Welfare: Basic Plan for the Promotion of Cancer Control. https://www.mhlw.go.jp/file/06-Seisakujouhou-10900000-Kenkoukyoku/0000196975.pdf.

[3] Lebel S, Maheu C, Lefebvre M (2014). Addressing fear of cancer recurrence among women with cancer: a feasibility and preliminary outcome study. J Cancer Surviv.

[4] Cantrell MA (2011). A narrative review summarizing the state of the evidence on the health-related quality of life among childhood cancer survivors. J Pediatr Oncol Nurs.

[5] Schiavone S, Jaquet V, Trabace L, Krause KH (2013). Severe life stress and oxidative stress in the brain: from animal models to human pathology. Antioxid Redox Signal.

[6] Klaunig JE (2018). Oxidative stress and cancer. Curr Pharm Des.

[7] Penson RT, Partridge RA, Rudd P, Seiden MV, Nelson JE, Chabner BA, Lynch TJ Jr (2005). Laughter: the best medicine?. Oncologist.

[8] Cella DF, Tulsky DS, Gray G (1993). The Functional Assessment of Cancer Therapy scale: development and validation of the general measure. J Clin Oncol.

[9] EuroQol Group (1990). EuroQol; a new facility for the measurement of health-related quality of life. Health Policy.

[10] Zigmond AS, Snaith RP (1983). The hospital anxiety and depression scale. Acta Psychiatr Scand.

[11] Oshio A, Abe S, Cutrone P (2012). Development, reliability, and validity of the Japanese version of Ten Item Personality Inventory (TIPI-J). Jpn J Pers.

[12] Iorio EL (2022). The BAP test and the global assessment of oxidative stress in clinical practice. http://www.medial.cz/content/files/medial/download/prospekty/HaD/2010_4_1_BAP_TEST_PRESENTATION.pdf#search=%27Eugenio+Luigi+Iorio++BAP+test%27.

[13] Alberti A, Bolognini L, Macciantelli D, Caratelli M (2000). The radical cation of N,N-Diethyl-para-phenylendiamine: a possible indicator of oxidative stress in biological samples. Res Chem Intermed.

[14] Carratelli M, Porcaro L, Ruscica M, De Simone E, Bertelli AA, Corsi MM (2001). Reactive oxygen metabolites and prooxidant status in children with Down's syndrome. Int J Clin Pharmacol Res.

[15] Celi P (2011). Biomarkers of oxidative stress in ruminant medicine. Immunopharmacol Immunotoxicol.

[16] Fukuda S, Nojima J, Motoki Y (2016). A potential biomarker for fatigue: oxidative stress and anti-oxidative activity. Biol Psychol.

[17] Cella D, Hahn EA, Dineen K (2002). Meaningful change in cancer-specific quality of life scores: differences between improvement and worsening. Qual Life Res.

[18] Lee YJ, Kim MA, Park HJ (2020). Effects of a laughter programme with entrainment music on stress, depression, and health-related quality of life among gynaecological cancer patients. Complement Ther Clin Pract.

[19] Daley AJ, Crank H, Saxton JM, Mutrie N, Coleman R, Roalfe A (2007). Randomized trial of exercise therapy in women treated for breast cancer. J Clin Oncol.

[20] Kenne Sarenmalm E, Mårtensson LB, Andersson BA, Karlsson P, Bergh I (2017). Mindfulness and its efficacy for psychological and biological responses in women with breast cancer. Cancer Med.

[21] van der Wal CN, Kok RN (2019). Laughter-inducing therapies: systematic review and meta-analysis. Soc Sci Med.

[22] Sarink FS, García-Montes JM (2022). Humor interventions in psychotherapy and their effect on levels of depression and anxiety in adult clients, a systematic review. Front Psychiatry.

[23] Vlachopoulos C, Xaplanteris P, Alexopoulos N (2009). Divergent effects of laughter and mental stress on arterial stiffness and central hemodynamics. Psychosom Med.

[24] Zander-Schellenberg T, Collins IM, Miché M, Guttmann C, Lieb R, Wahl K (2020). Does laughing have a stress-buffering effect in daily life? An intensive longitudinal study. PLoS One.

[25] Sugita M, Kapoor MP, Nishimura A, Okubo T (2016). Influence of green tea catechins on oxidative stress metabolites at rest and during exercise in healthy humans. Nutrition.

[26] Sharma P, Thapliyal A, Chandra T, Singh S, Baduni H, Waheed SM (2015). Rhythmic breathing: immunological, biochemical, and physiological effects on health. Adv Mind Body Med.

[27] Sugiura T, Dohi Y, Takase H, Yamashita S, Tanaka S, Kimura G (2011). Increased reactive oxygen metabolites is associated with cardiovascular risk factors and vascular endothelial damage in middle-aged Japanese subjects. Vasc Health Risk Manag.

[28] Schöttker B, Saum KU, Jansen EH, Holleczek B, Brenner H (2016). Associations of metabolic, inflammatory and oxidative stress markers with total morbidity and multi-morbidity in a large cohort of older German adults. Age Ageing.

[29] Martin RA (2001). Humor, laughter, and physical health: methodological issues and research findings. Psychol Bull.

[30] Weisenberg M, Raz T, Hener T (1998). The influence of film-induced mood on pain perception. Pain.

